# Translating a child care based intervention for online delivery: development and randomized pilot study of Go NAPSACC

**DOI:** 10.1186/s12889-017-4898-z

**Published:** 2017-11-21

**Authors:** Dianne S. Ward, Amber E. Vaughn, Stephanie Mazzucca, Regan Burney

**Affiliations:** 1 Department of Nutrition, Gillings School of Global Public Health, and Fellow, Center for Health Promotion and Disease Prevention, University of North Carolina at Chapel Hill, 2202 McGavran-Greenberg Hall, CB 7461, Chapel Hill, NC 27599-7461 USA; 2 Center for Health Promotion and Disease Prevention, University of North Carolina at Chapel Hill, 1700 Martin L. King Jr. Blvd., CB 7426, Chapel Hill, NC 27599-7426 USA; 3 Department of Nutrition, Gillings School of Global Public Health, University of North Carolina at Chapel Hill, 1700 Martin L. King Jr. Blvd., CB 7426, Chapel Hill, NC 27599-7426 USA

**Keywords:** Web-based, Children, Nutrition environment, Implementation

## Abstract

**Background:**

As part of childhood obesity prevention initiatives, Early Care and Education (ECE) programs are being asked to implement evidence-based strategies that promote healthier eating and physical activity habits in children. Translation of evidence-based interventions into real world ECE settings often encounter barriers, including time constraints, lack of easy-to-use tools, and inflexible intervention content. This study describes translation of an evidence-based program (NAPSACC) into an online format (Go NAPSACC) and a randomized pilot study evaluating its impact on centers’ nutrition environments.

**Methods:**

Go NAPSACC retained core elements and implementation strategies from the original program, but translated tools into an online, self-directed format using extensive input from the ECE community. For the pilot, local technical assistance (TA) agencies facilitated recruitment of 33 centers, which were randomized to immediate (intervention, *n* = 18) or delayed (control, *n* = 15) access groups. Center directors were oriented on Go NAPSACC tools by their local TA providers (after being trained by researchers), after which they implemented Go NAPSACC independently with minimal TA support. The Environment and Policy Assessment and Observation instrument (self-report), collected prior to and following the 4-month intervention period, was used to assess impact on centers’ nutrition environments. Process data were also collected from a sample of directors and all TA providers to evaluate program usability and implementation.

**Results:**

Demographic characteristics of intervention and control centers were similar. Two centers did not complete follow-up measures, leaving 17 intervention and 14 control centers in the analytic sample. Between baseline and follow-up, intervention centers improved overall nutrition scores (Cohen’s d effect size = 0.73, *p* = 0.15), as well as scores for foods (effect size = 0.74, *p* = 0.16), beverages (effect size = 0.54, *p* = 0.06), and menus (effect size = 0.73, *p* = 0.08), but changes were not statistically significant.

**Conclusions:**

Core elements of NAPSACC were effectively translated into online tools and successfully implemented by center directors. Results suggest that the online program may have retained its ability to drive change in centers’ nutrition environments using a streamlined, self-directed, and flexible implementation approach. Results need to be confirmed in a larger more definitive trial.

**Trial registration:**

NCT02889198 (retrospectively registered).

**Electronic supplementary material:**

The online version of this article (10.1186/s12889-017-4898-z) contains supplementary material, which is available to authorized users.

## Background

Early care and education (ECE) is recognized as an important setting for childhood obesity prevention initiatives because of the number of children these programs reach and the influence they have on children’s eating and physical activity behaviors [1, 2]. In the United States, center-based ECE programs alone provide care for a third of children under the age of 6 years [3], including many children from racial and ethnic minorities (non-Hispanic Black = 42%, Hispanic = 27%) and low-income families (below poverty = 23%) [3] who are at increased risk of obesity [4]. Public health initiatives to address childhood obesity are calling upon ECE programs to implement evidence-based strategies to promote healthier eating and physical activity habits in children [5, 6]. For example, ECE programs can encourage healthier dietary intakes by serving healthy foods, providing repeated food exposures, limiting access to unhealthy foods, providing healthy role models, and teaching children the knowledge and skills needed to make healthy food choices [7].

While the growing number of ECE-based intervention studies over the past decade offer many promising strategies to reduce childhood obesity [1, 8], adoption and implementation of these strategies into ECE settings has been limited [9]. Most ECE-based intervention studies to date have focused on establishing efficacy of different approaches [8], hence intervention delivery is tightly controlled and often directly administered by researchers. Translation of evidence-based strategies into real world settings can encounter barriers such as financial costs, time requirements, need for educational or training resources, and lack of comfort and/or behavioral capacity [10–13]. Research is greatly needed to better understand how evidence-based obesity prevention strategies can be effectively translated for community implementation in ECE programs.

Dissemination and implementation studies represent an important next step that will help advance ECE-based obesity prevention initiatives. Dissemination research examines the effectiveness of targeted efforts to distribute information and intervention materials to specific public health audiences on the knowledge about and use of evidence-based interventions [14]. Implementation research examines the impact of specific activities and strategies when trying to integrate evidence-based interventions into specific settings (e.g., ECE programs) [14]. While dissemination and implementation studies within ECE-settings are lacking, there is growing recognition of the need to address this gap [9].

The NAPSACC program, is a prime example of an evidence-based obesity prevention initiative designed for the ECE setting and recommended as an important approach for broader dissemination and implementation efforts [15–17]. NAPSACC was originally developed in 2002 to help ECE programs, specifically program administrators, improve their food and physical activity environments [18, 19]. The original intervention employs components of Social Cognitive Theory (e.g., expectancies, observational learning, self-efficacy, behavioral capacity, environment, situation, reinforcement, reciprocal determinism) [20] to introduce changes to the child care environment that will foster healthier habits in children enrolled. For example, NAPSACC encourages ECE programs to provide healthy foods and beverages, work with teachers to adopt healthy feeding practices, offer formal and informal education to children, and to adopt policies that reinforce good nutrition practices. The original delivery model was designed with implementation in mind [21], using an existing network of ECE technical assistance professionals and training them to become “NAPSACC consultants.” NAPSACC consultants recruited ECE program administrators (e.g., center director or owner) to participate and then guided them through NAPSACC’s five-step process for change: (1) self-assessment, (2) action planning, (3) education, (4) technical assistance, and (5) reassessment. To implement the program, NAPSACC consultants work with the program administrators because they are the key gatekeepers who control what is provided to children, expectations regarding teacher practices and interactions with children, and program policies – key elements of the child care environment that NAPSACC targets for change. Administrators are encouraged to engage other key stakeholders (e.g., teachers, parents) throughout the change process, but administrators lead the effort. NAPSACC’s original pilot as well as its larger evaluation demonstrated significant improvements in the nutrition and physical activity environments of NAPSACC centers compared to controls (10–15% increase over baseline scores, *p* ≤ 0.01) [22, 23]. Findings have been replicated in Arizona, California, Connecticut, Maine, and North Carolina [24–26]. NAPSACC has also been shown to have an impact on child weight and physical activity [25, 27].

While NAPSACC has been shown to be an efficacious program for improving ECE environments, its reliance on specially trained technical assistance professionals for dissemination and implementation has limited its widespread use. The existing network of Child Care Resource and Referral agencies, the primary providers of technical assistance for ECE programs nationwide, are only able to reach a small fraction (~20%) of existing programs [28]. This problem is exacerbated in rural areas due to long transportation times and fewer service providers [29]. Even when technical assistance professionals are available, they may lack specific training in child health and nutrition [30] needed to implement NAPSACC. Furthermore, the time required by technical assistance professionals to help ECE programs implement NAPSACC generally prohibits its use unless there is dedicated funding.

The purpose of this paper is to describe the translation of NAPSACC into an online format (known as “Go NAPSACC”), implementation of the new Go NAPSACC program by child care center directors, and a randomized pilot study evaluating its impact on centers’ nutrition environments. Specifically, we will examine the feasibility of this modified delivery model, built around online tools and with limited but targeted support from a local technical assistance professional, to determine if it is a viable strategy for broadening the dissemination and implementation of this program, particularly in resource-limited rural areas. The pilot will thus determine the feasibility of this delivery approach while focusing implementation efforts on a portion of Go NAPSACC’s content, specifically the child nutrition section, which in turn will inform the planning of a larger, definitive trial of the full program.

## Methods

### Translation of NAPSACC into Go NAPSACC

The translation process began with an update of Go NAPSACC content to ensure that it was current with the most recent policies, recommendations, and scientific literature. The content update expanded the number of best practice recommendations, which are divided into five modules: Child Nutrition, Breastfeeding & Infant Feeding, Physical Activity, Outdoor Play & Learning, and Screen Time.

When developing Go NAPSACC, core elements of the original program were retained, including its 5-step process for change and the related implementation strategies (as defined by Powell et al. [31]). Go NAPSACC’s suite of online tools guide ECE programs through this 5-step process for change and continuous quality improvement, including (1) self-assessment, (2) goal setting and action planning, (3) implementation, (4) education and training, and (5) re-assessment. An example of what this process might look like is provided in Appendix A. The self-assessment tool is an *audit with feedback*, allowing ECE program administrators to evaluate their current performance. The goal setting tool is meant to *promote adaptability*, giving ECE program administrators the flexibility to choose which areas they want to work toward improving in a way that meets their needs and priorities. The action planning tool guides ECE programs to *develop a formal implementation blueprint* that will allow them to accomplish the goals they have set. The tips and materials tool enables the *distribution of educational materials* that help ECE program administrators as they implement their action plan and address any identified needs to provide education to teachers, parents, and children. While these tools were available in original program, the online tools allowed us to address key barriers to implementation and integrate some additional implementation strategies.

One of the major barriers to implementation of the original program was the dependence on NAPSACC consultants to recruit ECE program administrators and to assist them with implementation. *Providing local technical assistance* was an important implementation strategy; however, the lack of available consultants, need for special training, and lack of funding for consultant time meant that NAPSACC was often not available or could only be supported in a few ECE programs. Thus, Go NAPSACC needed to provide *centralized technical assistance* through its online program so that ECE programs had the sense of having a virtual NAPSACC consultant. The program itself provided this sense of a virtual NAPSACC consultant by taking advantage of the online format and web technology and programming to offer a very interactive experience while navigating through the tools.

One of the additional implementation strategies afforded by the online tools was the ability to *tailor strategies*. Tailoring helps ensure that users receive the most appropriate and pertinent materials, which in turn increases the likelihood that the material will be read, retained, and perceived as personally relevant [32]. Go NAPSACC’s tailoring took into consideration the variety of ECE programs that exist, specifically around the type of program (center-based vs. home-based), ages of children served, and length of day. For example, half-day programs do not have as many opportunities in the schedule to provide meals and snacks or to offer active play opportunities; therefore, the self-assessment, goal setting, and action planning tools use adjusted criteria.

Another new implementation strategy afforded by the online tools was *facilitation*, a process of interactive problem solving and support designed to promote improvements [31]. Go NAPSACC’s programming is able to use previously supplied data to create an interactive experience for ECE programs that helps them identify needed improvements and offers prompts for the next steps toward change. For example, at the completion of the self-assessment, the program prompts a review of results and selection of goals. Presentation of results and goals are based on data supplied in the self-assessment to help the ECE program administrator see where he/she is doing well and where there is room for improvement. Potential goals are also presented to the ECE program administrator so he/she can choose to work toward goals requiring small or large changes. Once goals are selected, the program prompts creation of action plans. Once an action plan is finalized, the program offers quick links to relevant tips and materials based on the goal selected and action plan created.

Throughout the process to create Go NAPSACC, developers engaged ECE program administrators and technical assistance professionals to review online tools and content. More specifically, six people (mix of program administrators and technical assistance professionals from North Carolina) reviewed website wireframes using paper-based versions of web pages to evaluate ease of navigation. Draft tools were also reviewed by our Community Advisory Group, a mix of ECE program administrators, teachers, and technical assistance professional in North Carolina. A follow-up meeting with our Community Advisory Group (with 13 attendees) was used to gather reactions to revised wireframes and to refine language and content on these pages. A later meeting with the Community Advisory Group (with eight attendees) was used to review drafts of the online tools and to gather information about the types of resources needed for the tips and materials library. One-on-one testing was conducted with five people (mix of administrators and technical assistance professionals) to evaluate ability to navigate through the online tools using a think-aloud protocol (where people talk through their thoughts as they try to complete the requested tasks) [33]. Their feedback helped identify problems (e.g., when navigation was not clear) as well as opportunities for improvements (e.g., when additional functionality would be useful), which helped ensure that the final tools had good usability and utility. Table 1 below describes the final online tools and the translation and addition of implementation strategies afforded in Go NAPSACC.

**Table 1:** Description of the Go NAPSACC tools

Tool	NAPSACC and Original Implementation Strategies	Go NAPSACC’s Translation of Original Implementation Strategies	Go NAPSACC’s Additional Implementation Strategies
Self-Assessment	*Audit with feedback* An 87-item self-assessment evaluates ECE program’s current performance around nutrition and physical activity. Items are scored on a 4-point scale (1 = minimal practice, 4 = best practice). Layout of the paper tool allows easy identification of best practices being met (responses in the right-most column).	*Audit with feedback* Self-assessments allow the ECE program administrator to evaluate current practices in five key areas: breastfeeding and infant feeding (25 items), child nutrition (46 items), physical activity (23 items), outdoor play and learning (20 items), and screen time (13 items). The ECE program can complete all five or focus on only those areas of greatest interest. Original 4-point scoring is retained.	*Tailor strategies* Items are tailored for type of program (center vs. family child care home), ages of children served, full-time vs. part-time programs, etc. *Facilitation* Upon completion, feedback is given on each sub-section highlighting where the provider is doing a “great job” vs. “on your way” (needing improvement).
Goal Setting	*Promote adaptability* The NAPSACC Consultant reviews self-assessment results with the ECE program administrator and helps identify goals for improvement.	*Promote adaptability* Goal setting tool allows the ECE program administrator to identify goals for improvement.	*Tailor strategies* Presentation of possible goals shows the ECE program administrator where they can go after small changes or where they will need to invest more dedicated effort.
Action Planning	*Develop formal implementation blueprint* The NAPSACC Consultant helps the ECE program administrator develop an action plan for each goal selected.	*Develop formal implementation blueprint* Once goals are selected, step-by-step action plans are available that offer guidance for implementing changes.	*Facilitation* Action plans are pre-filled and specific to each goal. The ECE program can use as-is or edit it to fit his/her needs and resources. The tool prompts for a target completion date and identification of internal (e.g., parents, staff) and external (e.g., food vendor) sources of support.
Tips and Materials	*Distribution of educational materials* Slides and talking points for five educational workshops (child obesity, nutrition, physical activity, staff wellness, and working with families) are available, which NAPSACC Consultants can use to provide training to ECE program administrators. Additional resources have to be identified by the NAPSACC Consultant.	*Distribution of educational materials* An extensive library of resources is available and organized for easy navigation – sorted by topic area and type of resource. Types of resources include staff education, classroom activities, menu planning guidance, educational videos, policy templates, etc. Resources have been thoroughly vetted to ensure compliance with best practices.	*Tailor strategies/Facilitation* The ECE program administrator is directed to relevant tips and materials at the completion of each action plan. *Tailor strategies* The administrator can identify “favorites” which creates a shortcut on their tips and materials landing page linking to that resource.

### Evaluation of Go NAPSACC’s implementation

A randomized pilot study was conducted to evaluate the implementation and impact of Go NAPSACC. The classification of this study as a pilot aligns with the definition by Eldridge and colleagues in which “part of a future study is conducted on a smaller scale to ask the question whether something can be done, should we proceed with it, and if so, how” (pg. 8) [34]. The pilot focused specifically on evaluating the implementation and impact of the Child Nutrition module of Go NAPSACC, in preparation for a larger trial that would test all five modules. The primary goals of this pilot were to determine whether the online tools with minimal personal technical assistance (1) worked as a delivery model and (2) showed some evidence that it could retain the effectiveness demonstrated by the original program. Measures collected at baseline and post-intervention assessed change in centers’ nutrition environments, specifically food and beverages provided and teacher practices. Protocols have been reviewed by the Institutional Review Board at the University of North Carolina at Chapel Hill (IRB # 14–0931) and have been registered at clinicaltrials.gov (NCT02889198, retrospectively registered August 26, 2016).

#### Recruitment

Three local ECE technical assistance organizations serving six counties in North Carolina agreed to assist with recruitment and Go NAPSACC implementation. The counties targeted were largely rural and low-income areas, where resources are often limited. Local technical assistance (TA) providers helped identify potential child care centers and directors who might be interested in taking part in this study. Although the Go NAPSACC tools can be used by many different types of programs, only centers (not homes) were targeted in this evaluation effort. Hence, center directors were the program administrators who needed to agree to participate in the Go NAPSACC intervention and study. Based on lists generated from local TA providers, research staff followed up with center directors by phone to confirm interest and screen for eligibility. Eligible centers had to have children currently enrolled who were between 3 and 5 years and a quality rating of at least 2 stars (out of 5) or be faith-based (exempt from rating). The quality rating system in North Carolina allows centers to be recognized for the quality of care they provide. Rating considers factors like teacher education, in-service trainings, teacher-to-child ratios, and the child care environment. Centers that had participated in NAPSACC during the past 6 months were excluded. Interested directors from eligible centers signed a Memorandum of Understanding as an agreement of their participation. Recruitment was conducted between June and July of 2015.

#### Randomization

Centers were randomly assigned (1:1) to receive either immediate access (intervention arm) or delayed access (control arm) to Go NAPSACC. Prior to randomization, centers were stratified by county to ensure that each local agency would have half of its centers getting immediate access to the program and half getting delayed access. Stratification by county also helped control for any potential differences between these geographic areas and their technical assistance staff that might influence implementation. A list of enrolled centers was provided to the study statistician, who then randomized participating centers into either intervention or control using a permutated block approach (block size of two to ensure equity between arms). Results of randomization were shared with the study coordinator, who then informed participating centers. Randomization occurred in September 2015 (following baseline measures).

#### Go NAPSACC implementation model

While Go NAPSACC tools can be used directly by center directors, local TA providers offered a minimal level of support to facilitate implementation. This minimal support model included one in-person meeting with the center director to orient them to the Go NAPSACC tools (e.g., how to register for an account, complete a self-assessment, review results, set goals, create and customize action plans, navigate tips and materials). During this orientation, TA providers gave directors a step-by-step manual for how to use the program, which included screen shots from the website. Following this orientation, TA providers conducted brief monthly check-ins by telephone or email (e.g., inquire about progress, assess need for additional assistance, remind about project timeline). To prepare local TA providers for this role, they completed a four-hour training led by a Go NAPSACC program expert (during September 2015). The training addressed why childhood obesity is a problem (e.g., prevalence, immediate and short term health risks), the role of child care in development of children’s healthy weight (e.g., how provisions, practices, and policies at child care can influence children’s eating and activity habits, best practice recommendations from Go NAPSACC for promoting healthy habits in children), demonstration of the Go NAPSACC tools, and how to support using the online resources, making progress on action plans, and overcoming barriers to success.

Center directors were given 4 months to use Go NAPSACC. Implementation occurred between September 2015 and February 2016 (allowing for slight variation among centers for when they would start). This intervention period is slightly shorter than the 6 months provided by original NAPSACC; however, the efficiency of the online tools that could be accessed directly by center directors (instead of having to wait for in-person visits) provided the rationale for shortening the duration. For the purposes of this pilot, directors were instructed to focus on the area of Child Nutrition even though four additional areas are also available (Breastfeeding and Infant Feeding, Physical Activity, Outdoor Play and Learning, and Screen Time). The pilot thus benefited from the flexibility afforded by Go NAPSACC’s online tools, which allow the directors to focus on just one area at a time instead of tackling all areas at once. Given the expanded content included in Go NAPSACC and the four-month intervention period of the pilot, it also helped avoid overwhelming center directors with multiple self-assessments and options for goal setting and action planning. Directors also received a timeline for completing different steps of the change process. During the first month, directors attended the Go NAPSACC orientation, completed the Child Nutrition self-assessment, and selected goals. When it came to selecting goals, directors were encouraged to set 5–6 goals related to foods provided, beverages provided, and feeding practices. During the second month, directors were encouraged to complete action plans, initiate implementation, and to identify helpful tips and materials. Implementation activities continued through the third and fourth months. At the end of the fourth month, directors were encouraged to retake the self-assessment and evaluate their progress. As the key gatekeepers to child care centers, center directors were the primary participants. However, directors were encouraged to engage additional stakeholders throughout the process to help ensure an accurate assessment of current practices and identification of goals and development of action plans that staff would be willing to implement.

#### Outcome measures

The primary outcome for the pilot study was change in centers’ nutrition environments, which was assessed using the self-report version of the Environment and Policy Assessment and Observation (EPAO-SR) [35]. The EPAO-SR collects data from multiple sources using paper surveys, which are subsequently combined into a single assessment of the child care environment. The EPAO-SR components include a Center Director Questionnaire, Teacher Questionnaires, and a Policy Document Review. Reliability testing demonstrated day-to-day variation in things like foods and beverages served and teacher feeding practices (with ICCs of 0.06–0.60); however, reliability improved with multiple days of data capture (increasing ICCs to 0.20–0.86) [35]. Validity testing demonstrated generally good agreement between self-report and observation for foods and beverages served and nutrition policy (with correlations of 0.25–0.85), but lower agreement with teacher practices (correlations of 0.004–0.46) [35]. To maximize the quality of data, multiple days were assessed as recommended by the EPAO-SR developers [35]. While the original tool captures both nutrition and physical activity related content, only the items related to nutrition were used in this pilot. This same instrument was used for both baseline (July through September 2015) and follow-up data collection (March through May 2016).

The EPAO-SR Center Director Questionnaire asked about menus, food purchase and preparation, guidelines for foods brought from home, provision of nutrition education activities for parents, and the presence of written policies or general practices around nutrition. Directors also provided demographic information such as years of operation, receipt of child care subsidies, and number, ages, and race/ethnicity of children served.

Child Care Teacher Questionnaires in the EPAO-SR included a General Questionnaire and a Day-Specific Questionnaire. The General Questionnaire assessed the general nutrition environment at the centers, such as, availability of drinking water sources, presence of a fruit or vegetable garden, foods allowed for classroom celebrations, staff feeding practices (e.g., role modeling, praise and encouragement), staff training in nutrition, and staff self-efficacy for promoting healthy nutrition behaviors in children. The Day Specific Questionnaire assessed foods and beverages offered to children at meals and snacks, and staff mealtime behaviors across one day. As recommended by developers of the EPAO-SR, two teachers (each) completed the Day-Specific Questionnaire for 2 days to capture natural variability of practices within centers. Thus, two preschool teachers at each center were asked to complete questionnaires, each responding about a different two different days (ideally four consecutive days total). Results from these two reports for the 2 days were combined to reflect center practices.

For this study, the EPAO-SR Policy Document Review was completed by research staff (rather than self-reported) based on director-provided center policy documents (e.g., parent and staff handbooks, lesson plans). The Policy Document Review coded for the presence or absence of written policy statements about nutrition.

Information from these EPAO-SR components was utilized in scoring. First, individual item responses were used to derive variables assessing compliance with 42 nutrition best practices (BPs) [36]. Each nutrition BP variable was scored on a 4-point scale (0–3), where higher scores indicate closer compliance with best practice (a method used in NAPSACC’s original evaluation [22]). These nutrition BP variables were then sorted into one of seven environmental components: foods provided (12), beverages provided (5), feeding practices (8), feeding environment (8), menus (1), education and professional development (6), or policy (1). Environmental summary scores were calculated by averaging the scores from the relevant nutrition BP variables; hence, summary scores could range from 0 to 3. An overall nutrition score was also calculated by summing the seven environmental summary scores; hence overall nutrition scores could range from 0 to 21.

#### Process evaluation

The primary goals of the process evaluation were to explore the acceptability of this online delivery format and the potential time savings to TA providers. A sample of center directors from the intervention arm (*n* = 6) was interviewed at the end of the 4-month intervention period. From each geographic area (served by the local TA organization), one to four center directors were randomly selected to participate in these interviews. The exact number from each area was weighed so that the final sample interviewed represented proportionally the programs participating in the study. Semi-structured interview guides were developed to assess usability of the tools, ease of implementation, barriers encountered, and sufficiency of technical assistance. Interviews were recorded and each interviewer compiled a debriefing report on each of their interviews (revisiting recordings as needed) to summarize the major themes that emerged on each of the specified topics. In addition, each local TA provider was asked to keep a log of their Go NAPSACC implementation activities (date, length of contact, contents of discussion, assistance provided, etc.).

#### Statistical analysis

Impact of Go NAPSACC was assessed by comparing the difference in mean change in centers’ nutrition environment scores from pre- to post-intervention between centers randomized into the intervention arm (immediate access to GO NAPSACC) versus those randomized into the control arm (delayed access). Analyses used Generalized Linear Modeling (PROC GLM) and controlled for center characteristics that significantly predicted change (i.e., quality star rating, participation in the child and adult care food program (CACFP)). Cohen’s d effect sizes were also calculated to estimate the magnitude of the environmental changes, considering the small sample size of this study. The primary analysis examined all centers that completed pre- and post-intervention measures, but a secondary analysis examined only those completer centers where data were provided by the same teachers at both time points. This secondary analysis was conducted to limit the potential noise in the data that might arise from having different reporters at baseline compared to follow-up at individual centers. As noted above, the reliability testing of the EPAO-SR demonstrated some natural variations (day-to-day and between teachers).

#### Power and sample size

A power calculation was performed prior to the pilot study to determine sample size. The final calculation suggested that a sample size of 40 centers (20 per group) would have 80% power to detect a large effect size of 0.89, assuming an alpha of 0.05. Put into context, centers would need to make substantial improvement in at least five practices (increasing scores for each practice by 2 points, based on 4-point scales). Given that the assessment of the nutrition environment captured 42 practices, this effect size would require substantial improvement in at least 12% of practices.

## Results

### Sample characteristics

**Fig. 1:**
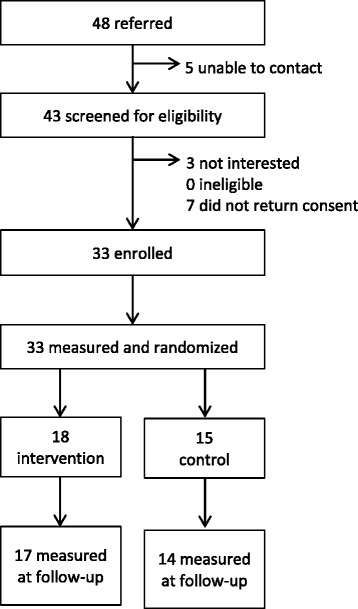
CONSORT Diagram for the Randomized Pilot of Go NAPSACC

Figure 1 illustrates participation by centers throughout the project (recruitment, screening, enrollment, baseline measurement and randomization, and follow-up measurement). Thirty-three centers signed a memorandum of understanding (MOU, organizational agreement to take part of the study) and participated in baseline measures (82.5% of the recruitment goal). Sample characteristics of the centers are presented in Table 2, but exclude one center which failed to return the Center Director Questionnaire which contained demographic information. On average, centers had been in operation for 16 years (the average was slightly higher in control vs. intervention centers, 19.3 vs. 13.2 years, respectively). Most centers were privately owned (84%) and had a quality rating of either 4 stars (41%) or 5 stars (34%). All but one center accepted subsidies, and the majority (75%) also participated in CACFP. Participation in subsidies and CACFP suggests that these centers serve many lower-income children. On average, these centers had 55 children enrolled, of which 22 were 3–5 years old. Of the preschoolers enrolled, there were large minority populations (67% Non-Hispanic Black, 11% Hispanic).

**Table 2:** Demographic characteristics of centers and children participating in Go NAPSACC pilot

	Total Sample (*n* = 32)^a^		Intervention (*n* = 18)		Control (*n* = 14)^a^	
	Mean	(SD)	Mean	(SD)	Mean	(SD)
Years in operation	15.8	(8.8)	13.2	(6.9)	19.3	(9.9)
Weekly enrollment fees	$132	(19.3)	$135	(18.0)	$128	(20.6)
	N	Percent	N	Percent	N	Percent
Type of center						
Privately owned	27	84.4%	15	83.3%	12	85.7%
Faith-based	8	25.0%	4	22.2%	4	28.6%
More at Four^b^	4	12.5%	4	22.2%	0	0.0%
Star Rating						
3	8	25.0%	4	22.2%	4	28.6%
4	13	40.6%	8	44.4%	5	35.7%
5	11	34.4%	6	33.3%	5	35.7%
Exempt (GS-110)	1	3.1%	0	0.0%	1	7.1%
Accepts subsidies	31	96.9%	17	94.4%	14	100.0%
CACFP participant	24	75.0%	14	77.8%	10	71.4%
NAEYC certified	1	3.1%	0	0.0%	1	7.1%
	Mean	(SD)	Mean	(SD)	Mean	(SD)
# children enrolled	54.9	(27.4)	61.8	(29.9)	45.9	(21.4)
# 3–5 year olds	22.2	(13.0)	23.8	(15.4)	20.1	(9.3)
	N	Percent	N	Percent	N	Percent
Race/Ethnicity of 3–5 year olds						
% Non-Hispanic Black	66.7	66.1%	66.1	55.3%	67.4	79.5%
% Hispanic/Latino(a)	11.2	11.5%	16.4	12.6%	5.0	5.8%

^**a**^One center did not report demographics

^b^More at Four is North Carolina’s pre-kindergarten initiative for at-risk 4-year-olds, designed to help prepare children for starting school

### Intervention impact on center nutrition environment

Two centers (one intervention and one control) failed to provide data at follow-up, resulting in 31 centers (17 intervention, 14 control) with sufficient data at both time points to be included in the analysis. The one intervention center without follow-up data was the same center that was missing the Center Director Questionnaire at baseline. Baseline environmental summary scores for these 31 centers showed only slight differences between those in the intervention versus control arm across the seven environmental components (ranging from 0 to 0.62). At follow-up, there was an increase in the overall nutrition score for intervention centers, but no change among control centers (+0.72 vs. +0.03, effect size of 0.73, *p* = 0.15). While the difference was not statistically significant and must be interpreted with caution, the medium to large effect size may suggest some level of practical importance in the change observed. Assessment of effect size is based on recommendations by Cohen [37]. Data are presented in Table 3.

**Table 3:** Baseline and follow-up nutrition environment scores from the EPAO-SR from centers participating in the Go NAPSACC pilot

	Intervention (immediate access)				Control (delayed access)				Intervention vs. Control			
	N	Baseline Mean (SD)^a^	Follow-up Mean (SD)	Effect size^b^	N	Baseline Mean (SD)	Follow-up Mean (SD)	Effect size	I - C^c^	Effect size	*p*-value	Adj* p*-value^d^
All Completers												
Overall nutrition score	17	9.48 (1.66)	10.19 (1.82)	0.43	14	10.06 (1.97)	10.09 (2.09)	0.02	0.69	0.73	0.06	0.15
Foods provided	17	1.78 (0.30)	1.96 (0.24)	0.69	14	1.82 (0.26)	1.86 (0.29)	0.13	0.15	0.74	0.06	0.16
Beverages provided	17	1.39 (0.36)	1.46 (0.26)	0.25	14	1.52 (0.26)	1.48 (0.33)	−0.16	0.12	0.54	0.16	0.06
Feeding environment	17	2.03 (0.27)	2.10 (0.25)	0.3	14	2.14 (0.28)	2.18 (0.27)	0.15	0.04	0.21	0.59	0.29
Feeding practices	17	0.99 (0.28)	0.97 (0.29)	−0.08	14	0.93 (0.30)	0.99 (0.35)	0.19	−0.08	−0.31	0.41	0.54
Menus	17	0.88 (1.27)	1.12 (1.32)	0.19	14	1.50 (1.56)	1.14 (1.46)	−0.25	0.59	0.73	0.06	0.08
Education and professional development	17	1.65 (0.68)	1.82 (0.69)	0.26	14	1.64 (0.49)	1.86 (0.62)	0.42	−0.05	−0.14	0.70	0.56
Policy	17	0.76 (0.44)	0.76 (0.44)	0.00	14	0.50 (0.52)	0.57 (0.51)	0.14	−0.07	−0.12	0.74	0.14
Restricted Sample^e^												
Overall nutrition score	17	9.44 (1.76)	10.20 (1.82)	0.44	13	9.95 (2.01)	9.97 (2.12)	0.01	0.75	0.80	0.05	0.12
Foods provided	17	1.78 (0.30)	1.96 (0.24)	0.69	13	1.81 (0.27)	1.84 (0.30)	0.11	0.15	0.77	0.05	0.22
Beverages provided	17	1.39 (0.36)	1.46 (0.26)	0.25	13	1.51 (0.27)	1.47 (0.34)	−0.14	0.12	0.51	0.19	0.06
Feeding environment	17	2.01 (0.31)	2.11 (0.24)	0.36	13	2.13 (0.30)	2.18 (0.28)	0.16	0.05	0.27	0.49	0.16
Feeding practices	17	1.00 (0.37)	0.98 (0.30)	−0.08	13	0.92 (0.31)	0.97 (0.36)	0.17	−0.08	−0.24	0.54	0.77
Menus	17	0.88 (1.27)	1.12 (1.32)	0.19	12	1.50 (1.57)	1.25 (1.54)	−0.17	0.49	0.63	0.12	0.19
Education and professional development	17	1.61 (0.71)	1.81 (0.71)	0.29	13	1.65 (0.50)	1.82 (0.63)	0.30	0.03	0.09	0.81	0.85
Policy	17	0.76 (0.44)	0.76 (0.44)	0.00	12	0.58 (0.51)	0.58 (0.51)	0.00	0.00	0.00	1.00	0.26

^a^SD = standard deviation

^b^Effect size = Cohen’s d effect size

^c^I – C = change in Intervention score – change in Control score

^d^ controlling for CACFP participation and quality rating

^e^ restricted sample included only those where data were coming from the same teacher at baseline and follow-up data collection

Similar findings were observed in three of the seven environmental summary scores with medium to large effect size but no statistical significance, specifically for menus (i.e., increase in variety of healthy foods on menu, effect size = 0.73, *p* = 0.08), foods provided (i.e., increase in healthy foods served, effect size = 0.74, *p* = 0.16), and beverages provided (i.e., increase in healthy beverages served, effect size = 0.54, *p* = 0.06). Also, a small to medium effect sizes was observed for feeding environment (effect size = 0.21, *p* = 0.29), but again this difference was not statistically significant. In menus, control centers appeared to start higher at baseline but then decrease (−0.36), while intervention centers started lower but increased (+0.24). For foods provided, intervention and control centers started out with similar scores at baseline; however, intervention centers increased (+0.18) while control centers remained stable (+0.04). The observed change in intervention centers seemed to come from improvements in amount of fruit served, type of fruit served, amount of dark green, orange, or yellow vegetables, and amount of lean meat served. For beverages provided, intervention centers appeared to start lower at baseline and increased slightly (+0.08), while control centers started higher but then decreased very slightly (−0.05). The observed change in intervention centers appeared to come from a decrease in the number of times sugary drinks are served, which was accompanied by increase in the control centers. For the feeding environment, intervention and control centers appeared to increase slightly (+0.08 and +0.04, respectively). The observed change appeared to come from an increase in intervention centers’ display of posters, books, and learning materials in classrooms. Again, these results, while interesting, should be interpreted with caution as none were statistically significant after adjustment.

An unexpected small to medium negative effect was observed for feeding practices (effect size = −0.31, *p* = 0.54). While feeding practice scores in each group remained fairly stable, this effect size resulted from a very slight decrease in scores across intervention centers (−0.02) and a slight increase in scores across control centers (+0.06). Specific nutrition BP variables (sub-components that went into the feeding practices score) where decreased scores were noted in intervention centers included requiring children to sit at the table until they cleaned their plate and use of a preferred food to encourage children to eat a less preferred food (lower scores on these items indicate they happened more frequently). However, intervention centers reported improvements in teachers asking children if they are still hungry before serving seconds. The only nutrition BP variable where control centers reported improvements was authoritative feeding (defined as a balance between encouraging children to eat healthy foods while also allowing children to make their own food choices).

The secondary analysis that was restricted to teachers who were present at both baseline and follow up strengthen findings in terms of the positive intervention effect on the nutrition environment (Table 3). Effect sizes for all content areas improved in favor of the intervention except menus, which slightly moved towards favoring control group. However, the environmental summary score for menus is calculated from only nutrition BP variable, so any minor changes that could have happened as a result of the decreased sample size when restricting to only repeat teachers would have a greater impact for this content area. Even in this restricted analysis, results did not reach statistical significance after adjustment.

### Program implementation

Interviews with a sample of center directors from the intervention arm suggested that Go NAPSACC was a very positive experience and few barriers were encountered. On average, directors rated their experience with Go NAPSACC very highly, rating it as 9 out of 10 (scale of 1–10, where 1 = very poor experience and 10 = wonderful experience, actual responses ranged from 8 to 10). One director noted “It gave me a lot of tips and a lot of stuff that I could do to help improve nutrition to help out with the children’s eating.” Similarly, another director noted “It was easy to do because it was online. And it gave me some great ideas…The whole thing gave me insight on the things to feed [the children].” Director interviews suggested that Go NAPSACC was working as intended, providing them with easy-to-use tools that guided them toward change. The self-assessment tool was seen as “self-explanatory” and was well-liked by all directors. As noted by one director, the self-assessment results “…really helped a whole lot. That made the process a lot easier because you saw right where you already were and then you could see where… it would be an easy fix to get to the next level… As far as seeing it and what to do to set the goals it was very user-friendly.” All directors recognized that the action planning tool positively impacted their ability to plan and reach goals, helping them think through the process for change and providing a resource they could refer back to as changes were being implemented. In addition, one director noted “Once you put it down, you’re making a commitment to do it. It kind of holds you accountable.” Many directors (4 of 6) also reported that the action planning tool helped them identify and engage others who could help them achieve their goals (e.g., parents, teachers, community). Directors also appreciated the tips and materials tool. As one director noted, “Everything was there that I needed. I didn’t have to go outside than what y’all had for us.”

While most of the director feedback was very positive, some barriers were still identified. The most commonly reported challenges were slow internet service, lack of computer literacy, and need for additional TA support. The slow internet service was not surprising given the rural areas in which these centers were located. The need for computer training was also expected, which is why an in-person orientation to Go NAPSACC was included in the implementation model. The experience was summarized by one director who noted “Once I got the training… it was pretty easy.” A need for ongoing TA was also anticipated, which is why the minimal support model was adopted instead of a no support model. While directors made note of these barriers, none appeared to prevent Go NAPSACC’s implementation.

The activity logs from TA providers showed good adherence to the minimal support model, and most TA providers felt that they were able to meet center directors’ needs for TA support. The largest commitment of time occurred during the first month, which coincided with the in-person orientation to the Go NAPSACC tool. Generally, the orientation took 45–60 min per center. Some directors were able to get through the orientation in as little as 15 min, while others took 90 min. After this first month, the time required for TA providers appeared to decrease. Generally, they followed up with centers 1–3 times per month by phone (even though the suggested protocol was for monthly check-ins), and the majority of calls were less than 30 min. The average time spent following up with centers after the initial orientation was just over an hour (71.8 min, range 20 min to 210 min). Additional in-person visits were rarely needed. Case example of how Go NAPSACC works is provided in Additional file 1.

## Discussion

Results of this study provide some initial evidence that Go NAPSACC has effectively translated core elements and implementation strategies from original NAPSACC into online tools and those tools may help center directors change their centers’ nutrition environments. Based on the small sample size, changes were not statistically significant. However, effect sizes were often medium to large, thus suggesting the practical importance of the changes observed. Medium to large effects were observed in overall nutrition score, foods provided, beverages provided, and menus; while a small to medium effect was observed for the feeding environment. The areas where greatest gains were observed overlap largely with the priority areas that directors were encouraged to address during goal setting (selecting 5–6 goals in foods provided, beverages provided, and feeding practices). The attention dedicated to improving foods and beverages served likely contributed largely to the changes in overall nutrition score and may have had a carryover effect to menus. The negative effect observed in feeding practices contradicted expectations. However, feeding practices may be subject to more variation. As noted in the results, observed changes around specific best practice compliance variables were mixed. Teachers at intervention centers were more likely to use some positive feeding practices (e.g., assessing child hunger before serving seconds) but also more likely to use negative practices (i.e., pressuring children to clean their plates, using food bribes to get children to eat less desired foods). Meanwhile, teachers at control centers also noted increased use of certain positive practices (e.g., authoritative feeding). It is possible that teachers in intervention centers adopted these negative practices in an attempt to get children to eat more fruits and vegetables (unknowingly adopting negative practices). It is also possible that teachers in control centers may have improved their practices in anticipation of the coming intervention. Go NAPSACC appears to have some promise in being able to make improvements in ECE nutrition environments, change which has proven difficult to achieve in other ECE-based implementation studies [38, 39]. However, a larger, more definitive, randomized control trial is needed to confirm these findings and conclusions and to evaluate the effectiveness in other content areas (e.g., modules on Physical Activity, Outdoor Play & Learning, and Screen Time) and across a broader age group of children (0–5 years old).

Go NAPSACC makes great strides toward addressing the major barrier to dissemination and implementation over the original NAPSACC model, namely the time and resources demands on TA providers. NAPSACC’s original implementation model required specially trained NAPSACC consultants to deliver in-person meetings, educational sessions, and multiple technical assistance contacts to all ECE programs. One NAPSACC study tracking TA provider time found that this model required approximately 25 h per center [25]. Even with this intense model of TA support, the changes observed at centers across studies have generally been modest, albeit significant [22, 23, 25]. The TA activity logs collected in the current pilot suggest that this time burden can be greatly reduced. A conservative estimate would be 5 h (1 h for orientation plus 1 h per month for check-in phone calls). However, even with this reduced TA, ECE programs still appear to be able to make improvements to the nutrition environment. Furthermore, online tools have the potential to reach ECE programs in areas not served by TA providers. Child Care Resource and Referral agencies, the primary providers of technical assistance for ECE programs, are only able to reach a fifth of existing ECE programs [28]. By comparison, 85% of the population, and even 80% of the population in rural areas, have internet access [40]. Thus, the Go NAPSACC model has the potential to reduce the effort required from TA providers to implement the NAPSACC program with the centers they already serve and to facilitate dissemination for the program to centers in rural areas with no or limited access to TA providers.

Go NAPSACC appears to have avoided potential challenges related to ECE providers’ lack of experience with computers and internet. While web-based interventions have shown great promise in changing individuals’ knowledge, attitudes, intentions, and behaviors across a wide array of health topics, including nutrition [41–43], it is unknown how well this will transfer to ECE programs given the limited attention received to date [44–46], particularly in programs related to child nutrition [47]. Some ECE technical assistance agencies may be hesitant to use online tools with ECE programs because they perceive ECE providers to have limited computer and internet skills. While there are no data available about technology use among ECE providers, it is at least perceived to be an issue. The concern is not unwarranted, as some directors in this pilot remarked on their lack of skills. However, these directors —even those with limited skills — were able to navigate the online tools and implement Go NAPSACC. Awareness of the target audience and their need for tools that were easy-to-use and intuitive helped Go NAPSACC avoid this potential barrier. Moving the program online, in turn, allowed for integration of additional implementation strategies, providing tailoring of strategies and facilitation. Additionally, ECE providers can work through tools at their own pace, at times that are most convenient for them, without waiting for visits from their TA providers.

The development and evaluation of the Go NAPSACC program had many strengths. Go NAPSACC offers a suite of well-designed, interactive tools that provide immediate and tailored feedback and guidance to ECE programs with limited assistance from a TA provider. Development carried forward core elements and implementation strategies from original NAPSACC, but also took advantage of technology to integrate additional implementation strategies. The pilot study, while small, used a rigorous randomized study design (with delayed access), employed a well-established measure of the ECE environment with good reliability and validity evidence, and collected measures at both a baseline and follow up.

The pilot nature of the evaluation did have some limitations, which should be addressed through a larger, and more definitive, randomized control trial. The small size of the final analytic sample was one limitation, as it was lower than recommended by the power calculation thus making it more difficult to see significant differences, despite the medium to large effect sizes observed. Recruitment and retention challenges contributed to this limitation. While TA providers referred sufficient numbers of centers to the study (often exceeding set goals of 10–20 centers), many of those centers did not end up enrolling (often because of difficulties contacting directors to confirm participation or because directors lost interest). While rural areas are an important target for interventions like Go NAPSACC that require low-resources in terms of outside support, recruitment goals may need to be lower and/or recruitment strategies enhanced. Screening strategies should also be enhanced to assess any prior participation in NAPSACC. While none of the centers had participated in NAPSACC during the last 6 months, any prior participation would be useful to aid interpretation of findings. Another limitation was that participating centers and directors were given only 4 months to implement Go NAPSACC. Changing teacher practices and education opportunities as well as creating new policies may require more time and additional resources. Go NAPSACC is intended to encourage continuous quality improvement in small and gradual steps, hence a longer period of time may have allowed for larger improvements. While 4 months likely provided sufficient time for completion of one cycle of the change process, future research should explore longer intervention periods encouraging multiple cycles to see if larger changes can be achieved, multiple content areas can be addressed, and whether or not changes are sustained. Another limitation was the measures used in the pilot. While they provide useful pilot data, more rigorous measures of Go NAPSACC effectiveness and implementation would be needed for the larger trial. In addition, assessment of cost effectiveness was not included in pilot, but would be important for the larger trial. A final limitation of the current pilot study was the limited incorporation of theory. Go NAPSACC’s underlying 5-step change process guides its implementation. However, there are several different types of theories, models, and frameworks that are useful to include in dissemination and implementation studies [48]. In a larger definitive trial, inclusion of a determinants framework like the Consolidated Framework for Implementation Research [49] could help assess contextual factors influencing implementation. Furthermore, inclusion of an evaluation framework like RE-AIM [50] could help guide a more rigorous process evaluation that would better assess reach, adoption, and implementation fidelity and incorporate more analytics from the online tools. Inclusion of theories like these in future work with a larger sample of centers is critical for answering questions regarding what types of centers participate in programs like Go NAPSACC (which is important for generalizability), how well programs are implemented, and what environmental factors influence implementation.

## Conclusions

This study demonstrates the successful translation of original NAPSACC into an online implementation model known as Go NAPSACC. The rigorous and thoughtful translation process that involved key stakeholders throughout was essential when trying to improve the efficiency of the delivery model to use web-based technology and encourage more self-directed use of tools. While the randomized pilot of Go NAPSACC suggested that the program retains its ability to have a positive effect on the nutrition environments in ECE centers, there is still much that can be learned from this program through future studies. Incorporation of implementation frameworks and theories into future studies would greatly inform our understanding of the contextual factors that may influence adoption and implementation. Future studies should also focus on a longer implementation period to allow for more rounds of quality improvement as well as the longer-term sustainability of the program. Also, training and resources focused on feeding practices should be considered, in light of the negative results for that subscale of the EPAO-SR. Overall, the Go NAPSACC program shows promise to be an effective, streamlined, self-directed, and flexible implementation approach for ECE programs to improve their nutrition environments.

## Additional file

Additional file 1: Case example of how the Go NAPSACC program works. (DOCX 15 kb)

## Abbreviations

BP: Best practices
CACFP: Child and adult care food program
ECE: Early care and education
EPAO-SR: Self-report version of the environment and policy assessment and observation
TA: Technical assistance
