# Supplementary material for: Translating a child care based intervention for online delivery: development and randomized pilot study of Go NAPSACC
Source: BMC Public Health. 2017 Nov 21;17:891. doi: 10.1186/s12889-017-4898-z (PMC5698966; doi:10.1186/s12889-017-4898-z)
Supplement: Additional file 1: — Case example of how the Go NAPSACC program works. (DOCX 15 kb) [file 12889_2017_4898_MOESM1_ESM.docx]

Additional File 1.

Case example of how the Go NAPSACC program works.

- The director uses the registration tool to create a Go NAPSACC account and registers her center. The registration tool captures program details, for example, that she runs a child care center and serves only children 2-5 years old.
- The director uses the self-assessment tool to evaluate her center’s nutrition-related practices (limited focus for this study). The Child Nutrition self-assessment includes all items, since questions focus primarily on feeding of toddlers and preschoolers (ages served by her center). The director is prompted to view results at the end of her assessment.
- The director reviews a summary of results from her Child Nutrition self-assessment and sees that she is doing well in terms of foods and beverages provided and menus, but still has room for improvement in terms of feeding environment, feeding practices, education and professional development, and policy. The results page prompts the director to move on to select specific goals.
- The director uses the goal selection tool to set priorities for changes. She decides to work on feeding practices and education. Specifically, she chooses the following goals:
  - Teachers always praise children for trying new or less-preferred foods
  - Teachers incorporate planned nutrition education into their classroom routines 1 time per week or more
- The director uses the action planning tool to create a step-by-step process for accomplishing the selected goals. For each goal, the tool generates a sample action plan. Each plan is broken down into several steps that encourage engagement of key stakeholders (e.g., teachers), gathering information and sharing it with key stakeholders, and checking in with stakeholders as new practices are being adopted, and celebrating success. She edits each of her plans to specify teachers and staff who can help her at each step. She also adjusts the anticipated completion date to allow herself two additional weeks to complete all of the steps.
- As suggested by the action plan, the director meets with teachers to talk about trying to improve practices around offering praise and education to children. During the meeting, she asks teachers about what resources they would need to help them change their current practices. She also brainstorms with teachers about key times in their daily routines where they might incorporate nutrition education.
- The director uses the tips and materials tool to find educational resources that she can share with her teachers about praise and education. She finds a video “Pass the Peaches” that describes how to create positive and pleasant mealtime environments. She also finds several storybooks and lesson plans about healthy eating that she can share with teachers. She shares these resources with teachers and asks them to use them to increase their use of praise and incorporate nutrition weekly nutrition education into their circle time activities (as discussed in the brainstorming session).
- The director revisits the action plans created to monitor completion of each step. This review prompts her to check back in with teachers to evaluate how changes are going.
- Teacher check-ins show that the changes are going well. Teachers are using praise more consistently and have successfully incorporated nutrition education into circle time at least once a week. Director makes sure to congratulate teachers for these improvements.
- Director retakes the Child Nutrition self-assessment to document these improvements and to identify priorities for future improvements.
